# Comparative evaluation of the diagnostic accuracies of four different malaria rapid diagnostic test kits available in Ghana

**DOI:** 10.1371/journal.pone.0302840

**Published:** 2024-05-07

**Authors:** Enoch Aninagyei, John Gameli Deku, Keren Trishia Yemofio, Ekua Quainoo, Kofi Adjei Ntiri, Evelyn Yaro, Priscilla Essandoh, Hubert Kwame Agbogli, Richard Harry Asmah

**Affiliations:** 1 Department of Biomedical Sciences, School of Basic and Biomedical Sciences, University of Health and Allied Sciences, Ho, Ghana; 2 Department of Medical Laboratory Sciences, School of Allied Health Sciences, University of Health and Allied Sciences, Ho, Ghana; 3 Ghana Health Service, Amamorley Health Center, Ga North Municipality, Greater Accra Region, Ghana; 4 Ghana Health Service, Ga North Municipal Health Directorate, Ofankor-Accra, Ghana; Addis Ababa University, ETHIOPIA

## Abstract

Malaria rapid diagnostic test (mRDT) kit is one of the techniques for diagnosing malaria. Due to its inherent advantages over the microscopy technique, several brands of the kit have flooded malaria endemic countries, without prior in-country evaluation. Two of such mRDT kits are *Oscar* (India) and *Standard Q* (Korea Republic). In this study, the performance of *Oscar* and *Standard Q* mRDT kits were compared to *First Response* (India) and *CareStart* (USA) mRDTs, which have been evaluated and deployed for use approved by the Ministry of Health (MOH). In this comparative study, whole blood samples were collected from patients suspected of malaria. *Plasmodium falciparum* was detected in each sample using nested polymerase chain reaction (nPCR), microscopy and the four mRDTs. The sensitivities, specificities, accuracies, positive and negative predictive values and accuracies of the mRDTs were determined using nPCR as a reference technique. Kappa statistic was used to determine the level of agreement among the techniques. Two hundred (200) blood samples were analyzed in this study. The overall detection rates of *P*. *falciparum* by microscopy, *First Response*, *CareStart*, *Oscar-PfHRP2*, *Standard Q* mRDT kits and nPCR were 31.5%, 34.5%, 33.5%, 32%, 31% and 43% (*x*^2^ = 6.1, p = 0.046), respectively. The accuracies of *CareStart* and *First Response* were comparable (90.5% vs. 89.5%). Further, comparing their sensitivities, *Oscar-PfHRP2* was 74.4% (95% confidence interval (CI): 63.9–83.2) while that of *Standard Q* was 72.1% (95% CI: 61.4–81.2), with comparable accuracies (*Oscar-PfHRP2*–89% and *Standard Q* -88%). Apart from *First Response* that was 98.3% specific, the others were 100% specific. Kappa test revealed perfect diagnostic agreement (κ = 0.90–0.98) among the four mRDTs. That notwithstanding, *Oscar-PfHRP2* agreed better with *CareStart* (κ = 0.94) and *First Response* (κ = 0.92) compared to the agreement between *Standard Q* and, *CareStart* (κ = 0.92) and *First Response* (κ = 0.90). Taken together, the diagnostic performance of the four mRDT kits were statistically similar. That notwithstanding, new mRDT kits should be evaluated prior to deployment for use.

## Background

Microscopy remains the gold standard for diagnosing malaria in endemic areas [1]. This is because the causative organism for malaria is directly observed during microscopy. In spite of the advantages of malaria microscopy, the technique has several limitations, including higher detection limits, being operator dependent, lack of quality assured reagents and long turnaround time [2,3]. To circumvent these demerits of microscopy, malaria rapid diagnostic testing (mRDT) kits emerged in the 1990s [4] into largely an unregulated markets. The performance of some of the kits was a major concern to users [5]. However, mRDT kits have gained popularity over the years due to their simplicity, high sensitivity, amenable to different testing conditions, short turnaround time and non-reliance on electricity [6]. As at 2019, there are over 200 different mRDT kits available globally and about 66% are found in sub-Saharan Africa [7]. Though there are variations among the mRDT kits, the parasite detecting principles are similar [7]. Irrespective of the brand manufacturers of the these kits, they detect either *Plasmodium falciparum* histidine-rich protein 2 (PfHRP2), *Plasmodium* aldolase and *Plasmodium* lactate dehydrogenase (pLDH) [8].

In Ghana, mRDT kits are procured and distributed by the MOH, through the National Malaria Elimination Program (NMEP). Kits supplied through the national programs are always evaluated and approved by independent bodies such as the Food and Drugs Authority (FDA) and the Ghana Standard Authority (GSA). Suitable products are then deployed for use through the Central, Regional and District medical stores. Currently, two mRDT kits, namely, *First Response* (India) and *CareStart* (USA) are being used for malaria diagnosis, throughout the country. These kits are not sold in the Ghanaian open market.

However, due to the user-friendliness of mRDT kits and irregular supply by the Government of Ghana [9], several kits are imported into the country without prior evaluation of their sensitivities. At the time of this study, *Oscar* (India) and *Standard Q* (Republic of Korea) were widely used in the country. There was no evidence that *Oscar* and *Standard Q* mRDT kits were supplied through the Government of Ghana, and the product package did not have any evidence to show prior validation by the regulatory agencies in Ghana. To ensure public health confidence in the use of these mRDT kits, this study evaluated and compared the diagnostic accuracies of the ‘unapproved’ kits to the MOH deployed kits, using nested polymerase chain reactions (nPCR) as a reference technique, in patients suspected of malaria. The nPCR was used as a reference technique due to its superior detection advantage over other malaria diagnostic methods [10].

## Methodology

### Study design and sources of the mRDT kits

This prospective cross-sectional study assessed the accuracies of four mRDT kits available in Ghana. Of the four mRDT kits, two (*First Response* and *CareStart*) are supplied to health facilities through the NMEP, after prior evaluation while the other two (*Oscar* and *Standard Q*) were bought from the open market. Table 1 shows the manufacturer details of the mRDT kits.

**Table 1:** The manufacturer details of the malaria rapid test kits.

Parameter	*First Response*	*CareStart*	*Standard Q*	*Oscar*
Country of origin	India	USA	Korea Republic	India
Manufacturer	Premier Medical Cooperation PVT Ltd	ACCESSBIO	S.D. Biosensor	Oscar Medicare PVT Ltd
Lot number	74134205	M019563	QML1020149	HMP2110001
Expiry date	August 2023	October 2023	October 2023	September 2023
Analysis time	30 minutes	20 minutes	15 minutes	15 minutes
Ghana Government or MOH approval insignia	Yes	Yes	No	No
ISO certification	No	No	No	Yes (ISO13485)
Buffer type	Combo	Combo	Combo	Combo
Pipette	Fixed volume	Fixed volume	Fixed volume	Fixed volume
*Plasmodium* antigen detected	PfHRP2	PfHRP2	PfHRP2	PfHRP2 and pLDH
Manufacturer indicated detection limit	Not available	Not available	Not available	Not available

MOH: Ministry of Health; PfHRP2: *P*. *falciparum* histidine-rich protein 2; pLDH: *Plasmodium* lactate dehydrogenase; ISO: International organization for standardization; USA: United States of America.

### Study sites, populations, sampling period and storage prior to analyses

Patients suspected of malaria were recruited from two rural and one urban health facilities in three different districts in the Greater Accra region of Ghana. The rural facilities were Obom (Ga Central municipal) and Mayera-Faase (Ga West municipal) Health Centers, and the urban facility was Taifa Polyclinic (Ga East municipal). Samples were collected from December 2021 –March 2022, under the ethics approval number GHS-ERC002/03/18, issued by the Ghana Health Service Ethics Review Committee. The samples used for this study were collected for a study supported by Malaria Genetic Epidemiology in the United Kingdom. The study sought to release an open dataset of *P*. *falciparum* genome variation in 20,000 worldwide samples. The findings of that study has since been published [11]. In that study, venous blood samples were randomly collected from patients suspected of malaria, with prior written consent. Adult patients provided self-written consent whilst in the case of minors (< 18 years), written consent was obtained from accompanying adults, after which child assent was sort from the minor (< 12 years). Suspected patient with malaria was defined as having fever, chills, general feeling of discomfort, headache, nausea, vomiting, diarrhea, abdominal pain, and/or muscle or joint pain. Study participants across all ages without prior antimalarial chemotherapy were drugs were selected. Patients that declined to partake in the study and patients too sick to provide relevant study responses were excluded from the study. Whole blood were collected in an ethylenediaminetetraacetic acid (EDTA) tubes, stored at 4°C whereas dried-blood spots (DBS), prepared with ~50 μL of the whole blood were stored at room temperature (25°C), until molecular analysis. Approval to re-use the archived samples were obtained from the University of Health and Allied Sciences Ethics Review Committee (UHAS-REC A.11 [24] 22–23). Analyses of archived samples (mRDT and PCR) were done from September 2022 –November 2022.

### Sample size determination

Using the Cochrane’s formula, N = $\frac{Z^{2}p(1-p)}{d^{2}}$, where n is the sample size, z the confidence level at 95% (standard value of 1.96), d the margin of error at 5% (standard value of 0.05), the sample size of 160 was arrived at, using 11.8% malaria RDT prevalence of malaria in the Greater Accra [12].

### Laboratory analyses

#### Malaria microscopy

With about 6 μL of whole blood, a thick smear was done, air-dried, and stained with 10% Giemsa stain for 10 minutes (min) [13]. Parasites were detected using the x100 oil immersion objective. In positive samples, parasites were quantified using the WHO protocol [14]. According to the protocol, parasitaemia was estimated by dividing the number of asexual parasites per at least 200 leukocytes and multiplied by estimated white blood cell count of 8000 cells/μL.

#### Malaria RDT testing

The manufacturer’s procedures for detecting *P*. *falciparum* specific histidine-rich proteins 2 (PfHRP2) proteins in the four mRDT kits were the same. Briefly, 5 μL venous blood were dropped on the sample column of the rapid test kit. Two drops of assay diluent were added to the buffer window of the kit. Results were read after 30, 20 and 15 min for *First Response*, *CareStart*, *Oscar* and *Standard Q*, respectively. For each mRDT kit, tests with two red lines at both the control and the test band was considered as positive whereas tests with only one red band at the control band was considered as negative. In the case of the *Oscar* mRDT, a band at the PfHRP2 column and a control band was deemed positive for *P*. *falciparum*. Only one line at the test band without one at the control band was an invalid test. All invalid tests were repeated [15]. The study focused on only *P*. *falciparum* because it causes almost 99% of malaria in Ghana [16].

#### Molecular detection of *P*. *falciparum* in the DBS

*Nucleic acid extraction using saponin-chelex method*. The DBS was cut into pieces and placed in 2.0 mL microcentrifuge tubes. Exactly, 1 mL of freshly prepared 0.5% saponin in 1× phosphate-buffered saline (PBS) was added to each tube to completely soak the filter paper. The set up was vortexed and incubated at 4°C overnight. After the overnight incubation, the microcentrifuge tube was centrifuged at 12000 revolutions per minute (rpm) for 2 min and the supernatant comprising saponin and the debris, evacuated by suctioning. One milliliter of 1×PBS was then added to each sample and centrifuged again at 12000 rpm for 2 min. The process was repeated until there was no heme (red color) seen in the sample tubes. Subsequently, 50 μL of 20% chelex suspension and 100 μL of nuclease free water was added to each sample and heated at 96°C for 10 min in order to detach the nucleic acids into the chelex solution. Finally, the set-up was centrifuged at 5000 rpm for 5 min and 120 μL of the supernatant containing the nucleic acids was transferred into a newly labelled microcentrifuge tubes. The nucleic acids were stored at -20°C until nPCR analysis.

*Amplification of P*. *falciparum small subunit rRNA (ssrRNA) gene*. *P*. *falciparum* ssrRNA gene was amplified through nPCR protocol. The first run primer set was rPLU6 (5’-TTAAAATTGTTGCAGTTAAAAC-3’) and rPLU5 (5’- CCTGTTGTTGCCTTAAACTTC-3’) and the second run nPCR primer set was rFAL1 (5’- TTAAACTGGTTTGGGAAAACCAAATATATT-3’) and rFAL2 (5’- ACACAATGAACTCAATCATGACTACCCGTC-3’) [17]. The nPCR reactions, for the first run was made up of 6.25 μL OneTaq Quick load 2x master mix, 0.25 μL each of 22 μM forward and reverse primers, 2 μL DNA template and nuclease free water to make 12.5 μL. The *Plasmodium* ssrRNA gene was amplified using the following condition; 24 cycles of 95°C for 1 min; 55°C for 2 min and 72°C for 5 min. The second run nPCR was made up of 10 μL of OneTaq Quick load 2x master mix, 0.2 μL each of 22 μM forward and reverse primers, 1 μL DNA template and nuclease free water to make 20 μL volume. The second run nPCR conditions were 35 cycles of 95°C for 1 min; 55°C for 2 min and 72°C, and 5 min final extension. A final band size of 205 base pairs (bp) was diagnostic of *P*. *falciparum*.

### Analysis of results

Both descriptive and inferential data analyses were done with SPSS Version 24 (Chicago, IL, USA). The demographic variables were presented as percentages. The association of the demographic variables with the malaria nPCR outcome was done with Chi square. Further, the detection rates of each testing methods was done by dividing the number of samples that were positive samples by the total number of samples analyzed. In addition, the sensitivities, specificities, accuracies, positive and negative predictive values and accuracies of the malaria rapid test kits were determined using the nPCR as reference. The Kappa inter-rater agreements of the techniques were rated as follows: 0.01–0.20 slight agreement, 0.21–0.40 fair agreement, 0.41–0.60 moderate agreement, 0.61–0.80 substantial agreement and 0.81–1.00 almost perfect or perfect agreement [18]. Regarding the inferential statistics, p < 0.05 was considered statistically significant.

## Result

### Characteristics of the study participants

Two hundred (200) archived blood samples, collected from the patients suspected of malaria, were analyzed. Majority of the samples were collected from Mayera-Faase Health Center (59.9%) and 55% of the samples were collected from females. The overall age range was 0 and 91 years with mean, modal and median ages being 22 years, 1 year and 18 years, respectively. First quartile to third quartile of the age distribution was 5–33 years, with interquartile range being 28 years. Majority of the study participants were over 14 years (53.5%). The other demographic details are presented in Table 2. Majority of the study participants were recruited from the outpatient departments (88.5%, 177/200) of the health facilities. The nPCR positivity was significantly higher among inpatients (72.2%, 13/18) compared to outpatients (40.7%, 72/177) (p = 0.02). The mean temperature was significantly higher among those confirmed with malaria (37.4°C ± 0.8) compared to those without (36.9°C ± 0.55) (t-test = 4.31, p <0.0001). The mean hemoglobin level for patients with malaria (11.8±2.5 g/dL) did not differ from those without (11.9 ± 2 g/dL) (t-test = 0.22, p = 0.818). Sixty-three samples (31.5%) yielded parasitemia ranging from 1068–283944 parasites/μL of blood, whilst in 137 (68.5%) of the samples, no parasites were detected by microscopy. Among the 137 samples found to be negative by microscopy, *First Response*, *CareStart* and *Oscar* mRDTs detected PfHRP2 in 6, 4 and 1 samples, respectively. However, *Standard Q* did not detect PfHRP2 in one sample that was positive by microscopy. The nPCR detected 23 (16.8%) of *P*. *falciparum* ssRNA genes from the 137 microscopy negative samples whilst in all the microscopy positive samples, *P*. *falciparum* ssRNA genes were detected (Table 3).

**Table 2:** Association of the demographic characteristics of the study participants with nPCR outcome.

Variable		nPCR results		
	Total (N = 200) n (%)	Positive (N = 86) n (%)	Negative (N = 114) n (%)	Chi statistic (p-value)
***Name of health facility***				6.2 (0.045)
** Mayera-Faase Health Center**	119 (59.9)	45 (37.8)	74 (62.2)	
** Obom Health Center**	60 (30)	27 (45)	33 (55)	
** Taifa Polyclinic**	21 (10.5)	14 (66.7)	7 (33.3)	
***Age (completed years)***				10.3 (0.006)
** < 5**	63 (31.5)	18 (28.6)	45 (71.4)	
** 5–14**	30 (15)	19 (63.3)	11 (36.7)	
** > 14**	107 (53.5)	45 (42.1)	62 (57.9)	
***Gender***				2.3 (0.128)
** Male**	90 (45.0)	44 (48.9)	46 (51.1)	
** Female**	110 (55.0)	42 (38.2)	68 (61.8)	
***Marital status***				0.7 (0.871)
** Below marital age (<18 years)**	98 (49)	41 (41.8)	57 (58.2)	
** Single**	36 (18)	15 (41.7)	21 (58.3)	
** Married**	61 (30.5)	27 (44.3)	34 (55.7)	
** Other**	5 (2.5)	3 (60)	2 (40)	
***Education level***				1.1 (0.551)
** None**	9 (4.5)	5 (55.6)	4 (44.4)	
** Below pre-school**	38 (19)	7 (18.4)	31 (81.6)	
** Pre-school**	8 (4)	3 (37.5)	5 (62.5)	
** Primary**	50 (25)	25 (50)	25 (50)	
** Junior high or middle school**	53 (26.5)	28 (52.8)	25 (47.2)	
** senior high school**	29 (14.5)	12 (41.4)	17 (58.6)	
** Tertiary**	13 (6.5)	6 (46.2)	7 (53.8)	
***Occupation***				2.1 (0.239)
** Farming**	9 (4.5)	4 (44.4)	5 (55.6)	
** Government employee**	18 (4)	5 (27.8)	13 (72.2)	
** Pensioner**	3 (1.5)	1(33.3)	2 (66.7)	
** Preschooler**	8 (4)	3 (37.5)	5 (62.5)	
**Pupil^a^**	52 (26)	33 (63.5)	19 (36.5)	
**Student^b^**	25 (12.5)	8 (32)	17 (68)	
**Trading^c^**	35 (17.5)	17 (48.6)	18 (51.4)	
**Tradesperson^d^**	25 (12.5)	10 (40)	15 (60)	
** Unemployed**	25 (12.5)	5 (20)	20 (80)	

Other = Divorced (n = 1) and widower (n = 4)

^a^A pupil is a school going children up to junior high school

^b^A student is a school going person above junior high school

^c^ A trader buys goods and sells them

^d^ A tradesperson is a skilled worker that specializes in a particular handiwork.

**Table 3:** Clinical features of the study participants.

		nPCR results		
Variable	Total n (%)	Positive n (%)	Negative n (%)	p-value
***Participant category***				7.74 (0.02)^1^
Out-patient	177 (88.5)	72 (40. 7)	105 (59.3)	
In-patient	18 (9.0)	13 (72.2)	5 (27.8)	
Antenatal patient	5 (2.5)	1 (20)	4 (80)	
***Temperature (*** ^***o***^ ***C)*** *				
Mean		37.4±0.80	36.9±0.55	4.31 (<0.0001)^2^
Median		37.1	36.9	
Mode		36.8	36.6	
Min–Max		36.6–40.1	36.1–39.5	
***Hemoglobin (g/dL)*** *				
Mean		11.8±2.5	11.9±2.0	0.22 (0.818)^2^
Median		12.3	11.9	
Mode		12.3, 14.2	11.3	
Min–Max		4.9–16.6	7.2–16.9	
***Parasitemia (/μL)***				
Negative	137 (68.5)	23 (16.8)	114 (83.2)	
< 10,000	33 (16.5)	33 (100)	0.0	
10,001–100,000	26 (13)	26 (100)	0.0	
> 100,000	4 (2)	4 (100)	0.0	

* Mean ± standard deviation

^1^p-value determined by Fisher exact test, chi statistic (p-value)

^2^p-value determined by T-test, t-statistic (p-value).

### Detection rates of malaria by testing methods

The detection rate of malaria microscopy was 31.5% (63/200). The rates obtained by the mRDT kits varied. The detection rates found for *First Response*, *CareStart*, *Oscar-PfHRP2* and *Standard Q* were 34.5% (69/200), 33.5% (67/200), 32% (64/200) and 31% (62/200) respectively. None of the samples tested by *Oscar* was positive for *Plasmodium* lactate dehydrogenase (pLDH). The highest detection rate was obtained by nPCR (43%) (Table 4). The detection rates obtained by the microscopy, mRDT kits and nPCR were significantly different (*x*^2^ = 6.1, p = 0.046). However, the performance of the mRDT kits were similar (*x*^2^ = 0.37, p = 0.831).

**Table 4:** Diagnostic performance of the laboratory techniques.

Technique	Positive n (%)	Negative n (%)
Microscopy	63 (31.5)	137 (68.5)
**mRDT kits**		
***First response***	69 (34.5)	131 (65.5)
***CareStart***	67 (33.5)	133 (66.5)
***Oscar***		
PfHRP2	64 (32)	136 (68)
pLDH	0 (0%)	200 (100%)
***Standard Q***	62 (31)	138 (69)
nPCR	86 (43%)	114 (57)

### The diagnostic efficiencies of mRDTs compared to the reference technique

Table 5 shows the comparative diagnostic performance of the mRDTs kits. Among the four mRDT kits, the sensitivities of *First Response* and *CareStart* were the same, 77.9% (95% confidence interval (CI): 67.7–86.1). However, *CareStart*, *Oscar-PfHRP2* and *Standard Q* were very specific (100%) while the specificity of the *First Response* was marginally lower (97.1%). On the other hand, the sensitivity of *Oscar-PfHRP2* (74.4%, 95% CI: 63.9–83.2) was marginally higher than that of *Standard Q* (72.1%, 95% CI: 61.4–81.2). The negative predictive values for *First Response* (85.5%) and *CareStart* (85.7%) were similar, while that of *Oscar-PfHRP2* (83.8%) and *Standard Q* (82.6%) were marginally lower. The accuracies of the kits ranged from 88%– 90.5%. Given the overlap between confidence intervals of the approved and unapproved kits, their accuracies were statistically similar.

**Table 5:** Diagnostic efficiencies of mRDT kits compared to nPCR.

		mRDT kits			
	Diagnostic variable	*First Response* *	*CareStart* *	*Oscar-PfHRP2*	*Standard Q*
**Diagnostic indices**	True positive	67	67	64	62
	True negative	112	114	114	114
	False positive	2	0	0	0
	False negative	19	19	22	24
	Sensitivity (95% CI)	77.9 (67.7–86.1)	77.9 (67.7–86.1)	74.4 (63.9–83.2)	72.1 (61.4–81.2)
	Specificity (95% CI)	98.3 (93.8–99.8)	100 (96.8–100)	100 (96.8–100)	100 (96.8–100)
	PPV^1^ (95% CI)	97.1 (89.4–99.3)	100 (96.8–100)	100 (96.7–100)	100 (96.8–100)
	NPV^2^ (95% CI)	85.5 (79.8–89.9)	85.7 (80.1–89.90	83.8 (78.3–88.1)	82.6 (77.2–86.9)
	Accuracy (95% CI)	89.5 (84.4–93.4)	90.5 (85.6–94.2)	89.0 (83.8–92.9)	88 (82.7–92.2)

* Ministry of Health (MOH) approved kits

^1^ PPV–Positive Predictive Value

^2^NPV–Negative Predictive Value.

### Diagnostic agreements between the diagnostic techniques

Table 6 details of the diagnostic agreements between the diagnostic techniques. All the four mRDT kits agreed substantially with the reference technique. However, among the mRDT kits, each agreed perfectly with the other. That notwithstanding, the Kappa agreement was stronger between *First Response* and *CareStart* (κ = 0.98). However, *Oscar-PfHRP2* agreed better with *CareStart* (κ = 0.94) and *First Response* (κ = 0.92) compared to the agreement between *Standard Q* and the approved kits.

**Table 6:** The inter-rater agreement between the mRDT kits and nPCR.

	nPCR	*First Response*	*CareStart*	*Oscar*
***First Response***	0.78 (89.5%)			
***CareStart***	0.80 (90.5%)	0.98 (99%)		
***Oscar-PfHRP2***	0.77 (89.1%)	0.92 (96.5%)	0.94 (97.5%)	
***Standard Q***	0.75 (88%)	0.90 (95.5%)	0.92 (96.5%)	0.93 (97%)

### Characteristics of the discordant mRDT kits results

Three same samples (Samples 1–3) were negative for *Oscar-PfHRP2* and *Standard Q*. The parasitemia range was 2338–3564 parasites/μL by microscopy. The samples were kept refrigerated in EDTA tube between 4°C for between 233–275 days. The patients were mildly (10.1 g/dL) to moderately (9.6 g/dL and 9.9 g/dL) anemic. *Standard Q* yielded two more discordant results than *Oscar-PfHRP2* did. Samples 4 and 5 was stored for almost similar (232–262) number of days before analysis. The hemoglobin levels (10.8 and 11.0 g/dL) of the patients with the malaria and parasitemia (2118 and 2740 parasites/μL) were also almost similar (Table 7).

**Table 7:** Sample characteristics and the nPCR results of the mRDT discordant results.

***First Response*/*CareStart*** positive and ***Oscar-PfHRP2*** negative					
Sample number	Collection date	Analysis date	Hemoglobin (g/dL)	Parasitemia (/μL)	nPCR
Sample 1	28/02/2022	03/11/2022	9.6	2338	Positive
Sample 2	15/03/2022	03/11/2022	9.9	3564	Positive
Sample 3	04/03/2022	03/11/2022	10.1	2740	Positive
***First Response*/*CareStart*** positive and ***Standard Q*** negative					
Sample 1	28/02/2022	03/11/2022	9.6	2338	Positive
Sample 2	15/03/2022	03/11/2022	9.9	3564	Positive
Sample 3	04/03/2022	03/11/2022	10.1	2740	Positive
Sample 4	16/03/2022	03/11/2022	11.0	2118	Positive
Sample 5	14/02/2022	03/11/2022	10.8	2740	Positive

## Discussion

This study independently assessed the detection rates of malaria using microscopy, *First Response*, *CareStart*, *Oscar-PfHRP2*, *Standard Q* mRDT kits and nPCR, among patients suspected of malaria. During the assessment, readers of each testing method was oblivious of the results of the other technique. The overall detection rates of *P*. *falciparum* by nPCR was significantly higher than that of microscopy, *First Response*, *CareStart*, *Oscar-PfHRP2* and *Standard Q* mRDT kits. In spite of the fact that the nPCR technique yielded the highest detection, it is not amenable to resource-limited laboratories, where malaria is mostly prevalent. Therefore, health facilities in such resource-limited settings depended on microscopy and mRDT kits for malaria diagnosis. However, mRDT kits is overtaking malaria microscopy as the method of choice for diagnosing malaria, especially at the outpatients departments and other screening points such as pharmacy shops. For this reason, several mRDT kits have flooded the Ghanaian market. Two of such kits are the *Oscar* and the *Standard Q* mRDT kits. Comparing the diagnostic indices of the Ministry of Health (MOH) deployed kits, the accuracies of the *CareStart* mRDT did not differ from the *First Response* kit. This is because; they were almost of equal sensitivity, specificity and accuracy. Moreover, the confidence intervals of the positive and negative predictive values overlapped.

Comparing *Oscar-PfHRP2* and *Standard Q*, they were of equal specificity with the same positive predictive values. However, *Oscar-PfHRP2* mRDT kit was slightly more sensitive, accurate with higher negative predictive value compared to *Standard Q*. Surprisingly, to the best of our knowledge, the performances of *Oscar-PfHRP2* and *Standard Q* mRDT kits have not been assessed in any published work. That notwithstanding, the performance of *Oscar-PfHRP2* and *Standard Q* mRDT kits agreed perfectly with that of *First Response* and *CareStart*, although, the Kappa agreement found for *CareStart* with the MOH approved kits was better than that of *Standard Q*.

The *Oscar-PfHRP2* and *Standard Q* mRDT kits yielded three and five discordant results respectively. It is not clear why the MOH-approved kits and nPCR detected *Plasmodium* antigens and genes in these samples but *Oscar-PfHRP2* and *Standard Q* did not. However, it must be noted that the samples were kept refrigerated in EDTA tube between 4°C for between 232–275 days. *Plasmodium* histidine-rich protein 2 (PfHRP2) may have been degraded in that samples and the remaining quantities were below the detection limits of the *Oscar-PfHRP2* and *Standard Q* mRDT kits. PfHRP2, like all other proteins, has been found to be unstable during storage [19]. The stability of proteins during storage is affected by various factors such as temperature [20], pH [21] and humidity [22]. According to a study, PfHRP2 proteins stored at -80°C in 50% glycerol remained stable for up to 6 months [23] and when stored at -20°C in 50% glycerol proteins remained stable for up to 12 months [23]. Therefore, the storage conditions for these five samples could have contributed to the protein degradation. Further, the samples containing parasites between 2118–3564 parasites/μL were collected from patients with malaria that were mildly or moderately anemic. The samples were stored in EDTA tubes. Given that EDTA causes hemolysis in relatively a short storage time [24], the parasite proteins were degraded during storage. EDTA cause irreversible structural, biochemical and functional damage to blood platelets and other cellular proteins [25]. For this reason, it is imperative that future diagnostic evaluation are done on freshly collected samples. Samples analysed in this study had to be stored for that long due to difficulties in *First Response* and *CareStart* for this study. To avoid biased due to day-to-day variabilities in the samples, it was essential that all the samples were analysed on the same day.

Comparing the manufacturer details of the mRDT kits, the testing time for *Oscar-PfHRP2* and *Standard Q* were relatively shorter compared to *First Response* and *CareStart*. Additionally, all the mRDT kits detect only PfHRP2, except *Oscar-PfHRP2* that have the added advantage of detecting *Plasmodium* lactate dehydrogenase (pLDH). With this additional advantage, *Oscar* mRDT kit can distinguish active *P*. *falciparum* infection from past infection, since pLDH is a marker for parasite viability [9,22,23]. Additionally, pLDH helps to detect parasites with PfHRP2 deletions [26] and finally, detect the presence of other parasites since the enzyme is present in all parasites [27].

The sensitivity of mRDTs have been shown in several studies to be of higher sensitivity compared to microscopy [28–30]. However, this study found the mRDTs to be only 0.5–3% more sensitive than microscopy. In the case of *Standard Q*, the sensitivity was 0.5% lower than that of microscopy. This observation is not farfetched since several factors affect the efficacy of mRDT kits. The clinical status of the patient is one factor where patients with asymptomatic, subclinical, submicroscopic or subpatent malaria are most likely to have false negative mRDT outcome compared to patients with clinical malaria [31]. This is because the higher the parasitemia, the better the performance of mRDT kits [32]. Further, malaria immunity conferred on individuals as a result of multiple or sustained exposure to the malaria parasite leads to depressed parasite multiplication [33], which can result in false negative mRDT test. In addition, the presence of antibodies against malaria antigens and antibodies *Mycoplasma pneumoniae* can interfere with mRDT performance, causing either false-positive or false-negative results [34]. Moreover, the age of the patient also affect the performance of mRDT kits. Children less than 5 years have either low or no immunity to malaria, and for that matter, malaria parasitemia could get to high densities in no time, leading to better mRDT kits performance [35–37].

In spite of the merits of mRDT kits, the PfHRP2-based kits have some limitations. The PfHRP2 protein could persist in the blood stream several weeks after malaria has resolved [38]. In addition, considering the emergence of non-falciparum *Plasmodium* in Africa [39] and specifically in Ghana [16], PfHRP2-based kits may yield false negative results. Finally, some of the malaria parasites have mutated their *hrp2* genes, making them unable to synthesis the HRP2 proteins [40] and parasites in Ghana are not exception. In a study published in 2022 by Duah-Quashie et al. [41], it was revealed that 30.7% and 17.2% of malaria parasites had their *pfhrp2* and *pfhrp3* genes, respectively deleted. It is for these reasons that mRDTs that detect both PfHRP2 and pLDH is preferred. Detection of the pLDH enzyme will not only indicate the presence of non-falciparum *Plasmodium* [42] but also parasite viability [43,44].

### Limitation

Samples analyzed in this study were stored in EDTA tube at 4°C for between 232–275 days. The prolonged storage period could affect the diagnostic performance of the kits. A larger sample size would have yielded a highly powered study.

## Conclusion

Given the overlap among the confidence intervals recorded for the kits, this study can conclude that *Oscar* and *Standard Q* performed statistically similar to *CareStart* and *First Response*. In spite of this observation, the accuracy of a newly introduced mRDT kits needs to be assessed prior to deployment for use.

## Abbreviations

FDA: Food and Drugs Authority
GSA: Ghana Standard Authority
PfHRP2: *Plasmodium falciparum* histidine-rich protein 2
ISO: International organization for standardization
mRDT: Malaria rapid diagnostic test
nPCR: nested Polymerase Chain Reaction
NMCP: National Malaria Control Program
NMEP: National Malaria Elimination Program
NPV: Negative Predictive Value
PLDH: Plasmodium lactate dehydrogenase
PPV: Positive Predictive Value
